# Targeting microRNA-23a to inhibit glioma cell invasion via HOXD10

**DOI:** 10.1038/srep03423

**Published:** 2013-12-05

**Authors:** Xing Hu, Dan Chen, Yanhui Cui, Zhiyuan Li, Jufang Huang

**Affiliations:** 1 Department of Anatomy and Neurobiology, School of Basic Medical Sciences, Central South University, Changsha, Hunan Province, China

## Abstract

Glioma is the most frequent primary brain tumor. Recently, the upregulation of microRNA (miR)-23a was found to be associated with glioma, but the molecular mechanism by which miR-23a promotes glioma growth remains to be unveiled. In the present study, we found that miR-23a was significantly upregulated in glioma tissues compared to their matched adjacent tissues. miR-23a was also highly expressed in glioma cell lines SHG44, U251, and U87 cells. Moreover, we identified homeobox D10 (HOXD10) as a novel target for miR-23a. The expression of HOXD10 was significantly reduced in glioma tissues and cell lines, and miR-23a negatively regulates the protein expression of HOXD10 in U251 and U87 cells. We further showed that miRNA-23a promoted U251 and U87 cell invasion, at least partially, by directly targeting HOXD10 and further modulating MMP-14. These findings suggest that miR-23a may serve as a promising therapeutic target for glioma.

Glioma is the most common primary brain malignancy, accounts for about 80% of cancers in the central nervous system, and is associated with a poor prognosis and high mortality^1^,^2^,^3^. Despite wide tumor excision combined with multi-agent chemotherapy and radiotherapy, the median survival rate of malignant glioma has not improved, and patients diagnosed with glioblastoma, the most malignant form of glioma, only live about 1 year after diagnosis^4^. Thus, the identification of novel therapeutic targets is critical for the treatment of glioma.

MicroRNAs (miRNAs) are small non-coding RNA molecules 18–25 nucleotides in length. It has been well established that miRNAs mainly inhibit gene expression by binding to the 3′ untranslated region (3′UTR) of their target genes to further suppress translation. Moreover, miRNAs were demonstrated to play critical roles in the development and progression of cancers and have been found to have positive or negative effects on cell proliferation, apoptosis, and invasion in various cancer cells^5^. There are various miRNAs associated with glioma, including miR-23a, miR-145, miR-155, miR-218, miR329, and so forth^6^,^7^. Recently, miR-23a has been indicated to be involved in the development and progression of multiple types of cancers, such as gastrointestinal cancer, colorectal cancer, esophageal squamous cell cancer, lung cancer, and glioma^8^,^9^,^10^,^11^,^12^. However, the detailed molecular regulatory mechanism of miR-23a in glioma cells remains unclear.

Homeobox D10 (HOXD10), a member of the Abd-B homeobox family, encodes a sequence-specific transcription factor with a homeobox DNA-binding domain^13^,^14^. Recently, several studies showed that the protein expression of HOXD10 was negatively regulated by miR-10b, an oncogenic miRNA in several cancers including glioma^15^,^16^. Whether other miRNAs are involved in the regulation of HOXD10 in glioma cells remains to be investigated.

In the present study, we demonstrated that the expression of miR-23a was significantly increased in glioma tissues when compared to the matched adjacent tissues. Three glioma cell lines, SHG44, U251, and U87, also showed higher expression of miR-23a. We further identified HOXD10 as a novel miR-23a target and found that HOXD10 was frequently downregulated in glioma tissues and cell lines. We also showed that miRNA-23a could induce U251 and U87 cell invasion, at least partially, via targeting HOXD10 and further modulating the expression of MMP-14, a crucial tumor invasion factor. In conclusion, our study indicated miR-23a could be a promising therapeutic target for glioma.

## Results

### The expression of miR-23a was upregulated in glioma tissues

**Figure 1:**
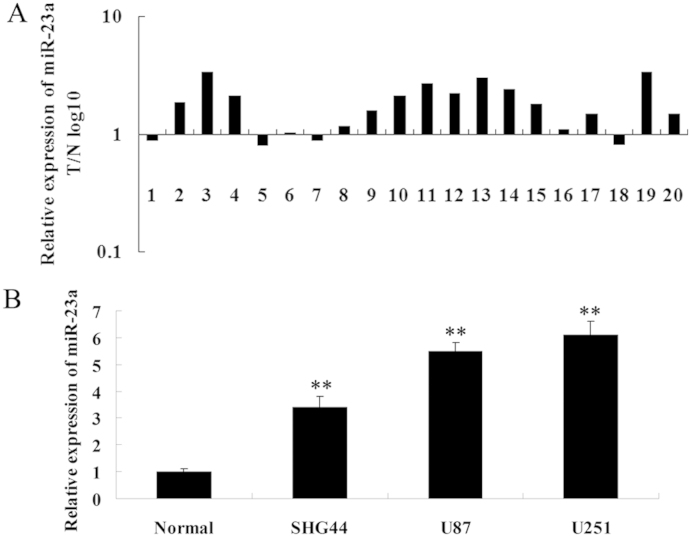
MiR-23a was upregulated in glioma tissues and cell lines. (A). Real-time RT-PCR was performed to determine the miR-23a level in 20 cases of glioma tissues and their matched adjacent normal tissues. It showed that miR-23a was frequently upregulated in glioma tissues compared to their matched normal tissues. T/N: tumor tissue/adjacent normal tissue. (B). Real-time RT-PCR was performed to determine the miR-23a level in non-tumor brain tissue and three glioma cell lines: SHG44, U251 and U87. Normal: non-tumor brain tissue. ** means P<0.01 vs. Normal.

We first determined the relative expression of miR-23a in 20 cases of glioma tissues and their matched adjacent tissues by real-time RT-PCR. We found that the miR-23a level was significantly upregulated in glioma tissues when compared to the matched adjacent tissues (Fig. 1A). We further examined miR-23a expression in three glioma cell lines: SHG44, U251, and U87. As shown in Fig. 1B, all these cell lines showed a positive expression of miR-23a that was higher than normal brain tissue. Based on these findings, we suggest that miR-23a upregulation may play a role in the development and progression of glioma.

### HOXD10 was identified as a novel miR-23a target

**Figure 2:**
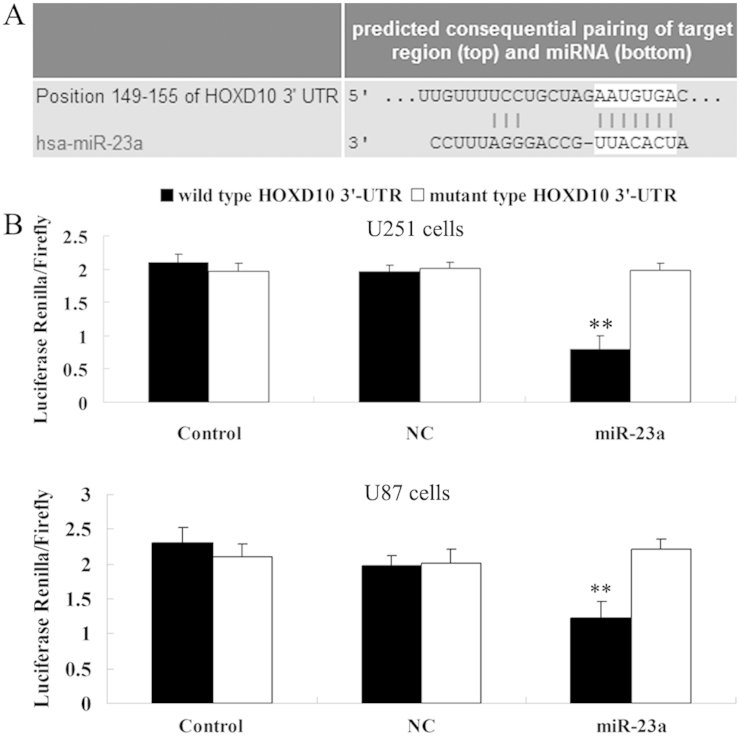
HOXD10 is a direct target gene of miR-23a. (A). Data of TargetScan showed the putative target sequence of miR-23a in the 3′-UTR of HOXD10. (B). Luciferase report assay data showed that co-transfection of U251 and U87 cells with miR-23a and wild type HOXD10 3′-UTR led to a remarkable decrease in luciferase activity. However, co-transfection with miR-23a and mutant HOXD10 3′-UTR had no effect on luciferase activity, and co-transfection with negative control (NC) miRNA and wild type HOXD10 3′-UTR or mutant HOXD10 3′-UTR also showed no difference. Control: U251 and U87 cells co-transfected with blank vector and wild type HOXD10 3′-UTR or mutant HOXD10 3′-UTR. ** means P<0.01 vs. Control.

Since bioinformatical analysis showed that HOXD10 was a putative target of miR-23a (Fig. 2A) and deregulation of HOXD10 was found to be associated with malignant tumors, we speculated that HOXD10 might be involved in the miR-23a mediated biological processes of glioma cells. Therefore, we conducted a luciferase reporter assay to clarify our speculation. As shown in Fig. 2B, U251 or U87 cells co-transfected with miR-23a mimic and wild type 3′-UTR of HOXD10 showed a notable decrease in luciferase activity compared to the control group (P<0.01). However, U251 or U87 cells co-transfected with miR-23a and mutant type 3′-UTR of HOXD10 showed no difference in luciferase activity compared to control groups. Those results indicate that HOXD10 is a novel target of miR-23a in glioma cells.

### The expression of HOXD10 was reduced in glioma tissues

**Figure 3:**
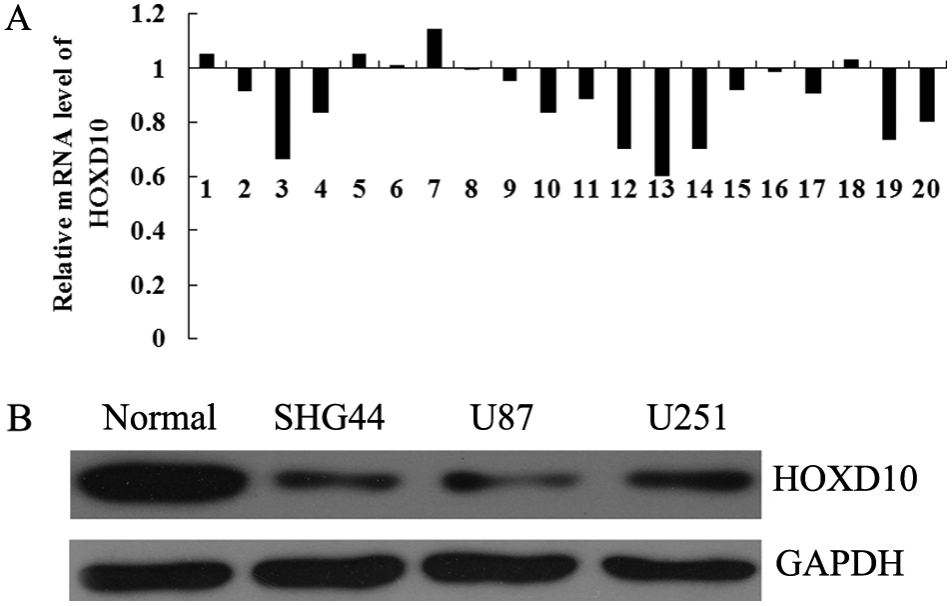
The expression of HOXD10 was reduced in glioma tissues. (A). Real-time RT-PCR was performed to determine the HOXD10 mRNA level in 20 cases of glioma tissues and their matched adjacent normal tissues. It showed that the mRNA level of HOXD10 was significantly reduced in glioma tissues compared to their matched adjacent normal tissues. T/N: tumor tissue/normal tissue. (B). Western blot was performed to determine the HOXD10 protein level in non-tumor brain tissues and three glioma cell lines: SHG44, U251 and U87. It showed that HOXD10 protein level was reduced in glioma cells compared to non-tumor brain tissues. Normal: non-tumor brain tissue.

We further examined the expression of HOXD10 in glioma tissues and their matched adjacent tissues, as well as in glioma cell lines. As shown in Fig. 3A, the expression of HOXD10 was frequently downregulated in glioma tissues compared to their matched adjacent tissues, which correlates with the expression of miR-23a and further indicates that HOXD10 is a target of miR-23a in glioma. Moreover, the protein level of HOXD10 was notably decreased in SHG44, U87, and U251 cells compared to non-tumor brain tissue as normal control (Fig. 3B). These findings suggest that HOXD10 may act as a tumor suppressor in glioma.

### The protein expression of HOXD10 was negatively regulated by miR-23a

**Figure 4:**
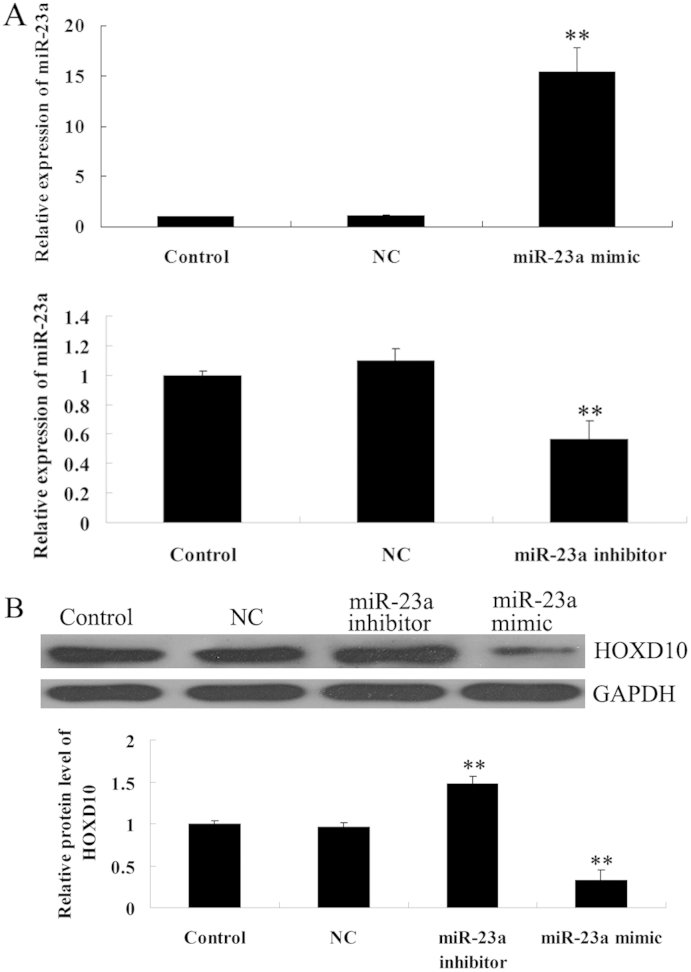
MiR-23a significantly downregulated the expression of HOXD10 in U251 cells. (A). Real-time RT-PCR was performed to determine the expression level of miR-23a after transfection of U251 cells with miR-23a mimic, miR-23a inhibitor, or scramble negative control (NC) miRNA. Control: cells without any transfection. ** means P<0.01 vs. Control. (B). Western blot was performed to determine the protein level of miR-23a after transfection of U251 cells with miR-23a mimic, miR-23a inhibitor, or scramble negative control (NC) miRNA. Control: cells without any transfection. ** means P<0.01 vs. Control.

To further investigate the effect of miR-23a on HOXD10 expression, we transfected U251 cells with scramble negative control (NC) miRNA, miR-23a mimic, or miR-23a inhibitor. As shown in Fig. 4A, the transfection efficiency was satisfactory. We then examined the protein level of HOXD10 in miR-23a-overexpressed or miR-23a-downregulated U251 cells. As shown in Fig. 4B, the protein levels of HOXD10 were downregulated in miR-23a-overexpressed U251 cells but upregulated in miR-23a-downregulated U251 cells when compared with that in the control group (P<0.01). These findings further confirmed that miR-23a negatively regulates the expression of HOXD10 in glioma cells.

### The roles of miR-23a and HOXD10 in the regulation of glioma cell invasion

**Figure 5:**
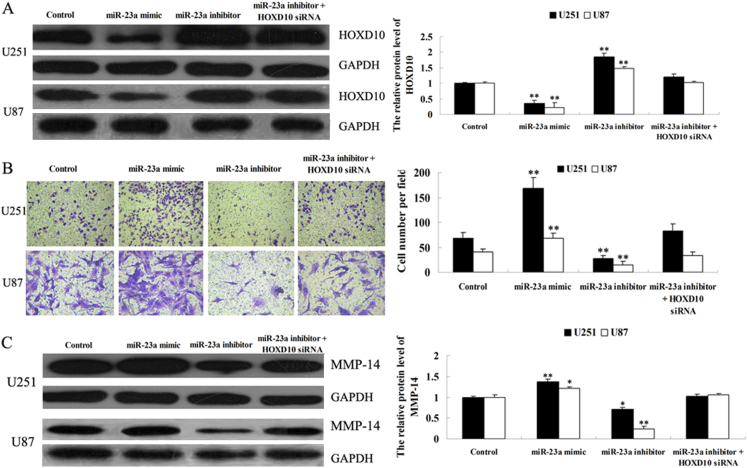
HOXD10 is involved in miR-23a-induced U251 and U87 cell invasion. (A). Western blot was performed to determine the protein level of HOXD10 in U251 and U87 cells transfected with miR-23a mimic, miR-23a inhibitor, or co-transfected with miR-23a inhibitor and HOXD10 siRNA. Control: cells without any transfection. ** means P<0.01 vs. Control. (B). Cell invasion assay was performed to determine the roles of miR-23a and HOXD10 in invasion for U251 and U87 cells transfected with miR-23a mimic, miR-23a inhibitor, or co-transfected with miR-23a inhibitor and HOXD10 siRNA. Control: cells without any transfection. ** means P<0.01 vs. Control. (C). Western blot was performed to determine the protein level of MMP-14 in U251 and U87 cells transfected with miR-23a mimic, miR-23a inhibitor, or co-transfected with miR-23a inhibitor and HOXD10 siRNA. Control: cells without any transfection. * means P<0.05 vs. control. ** means P<0.01 vs. control.

The roles of miR-23a and HOXD10 in glioma cell invasion were further investigated. After transfection of U251 and U87 cells with miR-23a mimic, miR-23a inhibitor, or co-transfection with miR-23a inhibitor and HOXD10 siRNA, we examined the protein level of HOXD10 using Western blot. As demonstrated in Fig. 5A, miR-23a had a negative effect on the HOXD10 protein level (P<0.01). Moreover, HOXD10 siRNA reversed the promoting effect of miR-23a inhibitor transfection on the levels of HOXD10 protein expression in U251 and U87 cells.

As shown in Fig. 5B, inhibition of miR-23a by transfection with miR-23a inhibitor suppressed U251 and U87 cell invasion, while upregulation of miR-23a promoted U251 and U87 cell invasion (P<0.01). Additionally, transfection of HOXD10 siRNA reversed the suppressive effect of miR-23a inhibitor transfection on U251 and U87 cell invasion. These findings suggest that miR-23a promotes glioma cell invasion, partially through targeting HOXD10.

Since HOXD10 has been shown to directly regulate the transcription of MMP-14, which plays a crucial role in cancer cell invasion, we further determined the protein expression of MMP-14 in U251 and U87 cells transfected with miR-23a mimic or miR-23a inhibitor, or co-transfected with miR-23a inhibitor and HOXD10 siRNA. As shown in Fig. 5C, miR-23a overexpression upregulated MMP-14 protein expression (P<0.05), and downregulation of miR-23a inhibited MMP-14 protein expression (P<0.01). Furthermore, HOXD10 downregulation by siRNA reversed the inhibitory effect of the miR-23a inhibitor on MMP-14 protein expression. Based on these findings, we suggest that miR-23a promotes glioma U251 cell and U87 invasion, probably by the regulation of MMP-14 via directly targeting HOXD10.

## Discussion

The present study showed for the first time a novel molecular mechanism of miR-23a and HOXD10 in glioma cell invasion. We found that miR-23a was frequently upregulated in glioma tissues and positively expressed in glioma SHG44, U251, and U87 cells. HOXD10 was identified as target of miR-23a, and the expression of HOXD10 was frequently reduced in glioma tissues. Moreover, miR-23a significantly suppressed the protein expression of HOXD10 in glioma cells. More importantly, we found that miR-23a induced glioma cell invasion by modulating MMP-14 via its target HOXD10.

Increasing evidence demonstrates that miR-23a acts as an oncogenic miRNA in various cancers. For instance, Jahid et al. reported that miR-23a promoted the transition from indolent to invasive colorectal cancer^17^. Liu and colleagues showed that miR-23a suppressed paclitaxel-induced apoptosis, promoted cell viability, and facilitated the colony formation ability of gastric adenocarcinoma cells^18^. However, evidence on the role of miR-23a in glioma was limited.

Recently, miR-23a was reported to have critical implications in glioma. Rao et al. performed a large-scale, genome-wide miRNA expression profile of 26 glioblastomas, 13 anaplastic astrocytomas, and 7 normal brain tissue samples^19^. It showed miR-23a was frequently upregulated in glioblastoma and anaplastic astrocytoma tissues, and inhibition of miR-23a downregulated the colony formation ability in malignant astrocytoma cells^19^. In our study, we showed that miR-23a was upregulated in glioma tissues, consistent with the findings from Rao and colleagues^19^. However, Yang and colleagues performed a genome-wide serum miRNA analysis and showed that miR-23a was significantly decreased in patients with grade II–IV astrocytoma^20^. Thus, these studies indicate that miR-23a has a different expression profile between glioma tissue as well as serum.

Several studies have focused on the molecular mechanism of miR-23a involvement in the regulation, development, and progression of glioma. Tan et al. reported that cAMP response element-binding (CREB) promoted the tumorigenesis of glioma via regulating the transcription of miR-23a and that miR-23a could directly target a key tumor suppressor PTEN, which is frequently silenced in glioma^21^. Therefore, miR-23a acts as a key modulator in CREB/PTEN regulated gliomagenesis. Wu et al. suggested that two clusters of miR-23a ~ 27a ~ 24 − 2 and miR–23b ~ 27b ~ 24 − 1 promoted glioma cell proliferation via cooperative regulation of MXI1^22^, which further confirmed the promoting effect of miR-23a on glioma cell proliferation. Moreover, Lian and colleagues showed that miR-23a expression was higher in glioma tissues of 79 samples than in the matched adjacent tissues, consistent with our findings, and that inhibition of miR-23a could suppress glioma cell growth via targeting apoptotic protease activating factor-1 (APAF1)^12^. Since miR-23a targets many genes in the same way, the other target genes of miR-23a in glioma as well as their function should be further investigated. We identified HOXD10 as a novel target of miR-23a and found that miR-23a inhibited glioma cell invasion at least partially by inhibiting the expression of HOXD10. Our study expands the understanding of the miR-23a molecular mechanism in the regulation of glioma.

HOXD10 has been demonstrated to play an important role in cell differentiation and morphogenesis during development^14^. Accumulating evidence suggests that HOXD10 also acts as a tumor suppressor in human malignancies. For instance, Wang et al. reported that through promoter hypermethylation, the expression of HOXD10 was significantly downregulated and associated with gastric carcinogenesis^23^. Moreover, HOXD10 was reported to be a direct target of miR-10b, which also acts as an oncogenic miRNA in glioma like miR-23a. Sun and colleagues showed that miR-10b induced glioma cell invasion by modulating MMP-14 and uPAR expression through directly targeting HOXD10 15. In our present study, we suggest a similar regulatory mechanism of miR-23a promoting glioma cell invasion via downregulating HOXD10, which further upregulates the expression of MMP-14, a key factor implicated in extracellular matrix breakdown in physiological or pathological processes. Since MMP-14 is overexpressed in neuroblastoma and closely associated with invasive depth and distant metastasis, our findings suggest that targeting miR-23a may effectively inhibit glioma invasion and metastasis, probably through MMP-14 pathway, however, further studies are warranted to confirm the connection between miR-23a and MMP-14 signaling cascade.

In conclusion, the present study showed that miR-23a was upregulated in glioma. This overexpression promoted glioma cell invasion, probably by modulating MMP-14 via directly inhibiting the expression of HOXD10, which was identified as a novel target of miR-23a. Based on these findings, our study suggests miR-23a may serve as a potential target for an effective therapeutic strategy to suppress glioma invasion and metastasis.

## Methods

### Reagents and materials

Dulbecco's Modified Eagle Media (DMEM), fetal bovine serum (FBS), TRIZOL agent, miR-23a mimics, miR-23a inhibitor, TaqMan MicroRNA Assay Kit, and Lipofectamine 2000 were purchased from Life Technologies (Carlsbad, CA, USA). PrimeScript RT reagent kit and SYBR Premix Ex Taq II were purchased from TaKaRa (Dalian, Liaoning, China). MiRNeasy Mini Kit was purchased from Qiagen (Valencia, CA, USA). Protein Assay Reagents were purchased from Bio-Rad Laboratories (Hercules, CA, USA). QuikChange Site-Directed Mutagenesis Kit was purchased from Stratagene (La Jolla, CA, USA). Mouse anti-HOXD10 monoclonal antibody, mouse anti-MMP-14 monoclonal antibody, mouse anti-GAPDH monoclonal antibody, and rabbit anti-mouse secondary antibody were purchased from Abcam (Cambridge, UK). PsiCHECK2 Vector and Dual-Luciferase Reporter Assay System were purchased from Promega (Madison, WI, USA). Cell Invasion Assay Kit was purchased from Merck Millipore (Darmstadt, Germany).

### Tissue specimen collection

The Ethics Committee of Xiangya Hospital of Central South University, Changsha, Hunan, China, approved all protocols in this study. All written informed consent was obtained from patients with glioma. Twenty primary glioma tissues and their matched adjacent tissues, as well as eight non-tumor brain tissues, were collected at the Department of Neurosurgery, Xiangya Hospital of Central South University, Changsha, China, from July 2012 to December 2012. Non-tumor brain tissue samples were obtained from 8 patients without malignancy, and they were collected by partial resections of normal brain tissue in order to reduce increased intracranial pressure when treating for the severe head injury. The tumor tissues were carefully dissected from the surrounding benign tissues with the help of a board certified pathologist, and the margin was confirmed through immunohistochemistry by two independent pathologists at the Department of Pathology, the Xiangya Hospital of Central South University. The surrounding non-tumor tissues were used as control for each tumor tissue. Those are deidentified samples, and no patient information is involved. Additionally, no patients had received blood transfusion, radiotherapy, or chemotherapy before surgery. All samples were immediately frozen in liquid nitrogen after surgical removal until used for experimentation.

### Cell culture

Three human glioma cell lines, SHG44, U251, and U87, were obtained from American Type Culture Collection (ATCC, Rockville, MD). Cells were cultured in DMEM added with 10% FBS, 100-U/ml penicillin, and 100 mg/ml streptomycin in a humidified atmosphere containing 5% CO_2_ at 37°C.

### RNA extract and quantitative real-time PCR (qRT-PCR)

For the mRNA expression assay, total RNA was extracted from tissues and cells using TRIZOL. Reverse transcription PCR was performed using PrimeScript RT reagent kit according to the manufacturer's instruction. After cDNA was obtained, real-time PCR was performed by SYBR Premix Ex Taq II. HOXD10-specific primer sequences were as follows: 5′- GACATGGGGACCTATGGAATGC -3′ (forward) and 5′- CGGATCTGTCCAACTGTCTACT -3′ (reverse). GAPDH was used as an endogenous reference, and GAPDH-specific primer sequences were as follows: 5′- ACAACTTTGGTATCGTGGAAGG -3′ (forward) and antisense primer 5′- GCCATCACGCCACAGTTTC -3′ (reverse).

For the miRNA expression assay, miRNAs were isolated by miRNeasy Mini Kit, and the miRNA expression was determined using the TaqMan MicroRNA Assays kit on a 7500 Fast Real Time PCR System (Applied Biosystems, Carlsbad, CA, USA) in accordance with the manufacture's instruction. U6 was used as an endogenous reference.

For each sample, independent experiments were repeated three times. The relative expression levels of mRNA and miRNA were analyzed by use of the 2^−ΔΔCt^ method.

### Transfection

For transfection, 1 × 10^5^ cells were harvested, seeded in a 24-well plate, and cultured for 24 h. According to the manufacture's protocol, miRNA mimic, miRNA inhibitor, scramble negative control miRNA, or eukaryotic expression plasmids were transfected into cells using Lipofectamine 2000. Cells were then cultured in a humidified atmosphere containing 5% CO_2_ at 37°C for 6 h, and then the transfection media was replaced by complete medium. After incubation for 48 h, other assays were performed using these cells.

### Luciferase reporter assay

The full length 3′-UTR of HOXD10 was cloned into the psiCHECK2 luciferase reporter vector. The QuikChange Site-Directed Mutagenesis Kit was used to generate a mutant 3′-UTR of HOXD10, which had a substitution of UGU to CAC within the miR-23a binding seed region. In accordance with the manufacturer's instruction, Lipofectamine 2000 was used to co-transfect miR-23a mimic with psiCHECK2 vector inserted with wild type or mutant type 3′UTR of HOXD10, into U251 and U87 cells, respectively. After incubation in a humidified atmosphere containing 5% CO_2_ at 37°C for 48 h, luciferase reporter assay was performed by the Dual-Luciferase Reporter Assay System according to the manufacturer's instruction. Each assay was performed in triplicate. Renilla luciferase was used for normalization.

### Western blotting

Cold RIPA lysis buffer was used to solubilize tissues and cells. Protein Assay Reagents were used to determine the protein concentration. Protein was then separated with 12% SDS-PAGE and transferred to a PVDF membrane which was then blocked in 5% nonfat dried milk in PBS at room temperature for 4 h. The membrane was then incubated with mouse anti-HOXD10 monoclonal antibody (1:200), mouse anti-MMP-14 monoclonal antibody (1:400), or mouse anti-GAPDH monoclonal antibody (1:400) at room temperature for 3 h. The membrane was then incubated with rabbit anti-mouse secondary antibody (1:40000) for 40 min. Enhanced chemiluminescence reagent detected the signal on the membrane. Data was analyzed by densitometry using Image-Pro plus software 6.0 and normalized to GAPDH expression. The gel blots shown in the figures were cropped for better presentation, the original whole gel blot were included in the supplementary data.

### Invasion assay

According to the manufacture's instruction, cell suspension containing 5 × 10^5^ cells/ml was prepared in serum free DMEM. For the invasion assay, 500 μl of DMEM with 10% FBS was added into the lower chamber, and 300 μl of cell suspension was added into the upper chamber. After incubation in a humidified atmosphere containing 5% CO_2_ at 37°C for 24 h, those non-invading cells and the extracellular matrix gel was gently removed from the interior of the inserts using a cotton-tipped swab. Invasive cells on the lower surface of the membrane were stained for 20 min, rinsed by water, and dried in air. Five fields were randomly selected under the microscope, and the number of cells through the membrane was counted.

### Statistical analysis

All data was represented as the mean of at least triplicate samples ± standard deviation. Statistical analysis included One-way ANOVA or Student's t test using SPSS 19.0. P values less than 0.05 were considered statistically significant.

## Author Contributions

X.H., J.H., and Z.L. designed the study. X.H., D.C. and Y.C. collected and analyzed the data. X.H., J.H. and Z.L. wrote the main manuscript and prepared all figures. All authors reviewed the manuscript.

## Supplementary Material

Supplementary Information Supplementary Data
