# Supplementary material for: Targeting microRNA-23a to inhibit glioma cell invasion via HOXD10
Source: Sci Rep. 2013 Dec 5;3:3423. doi: 10.1038/srep03423 (PMC3851882; doi:10.1038/srep03423)
Supplement: Supplementary Information — Supplementary Data [file srep03423-s1.pdf]

# Supplementary Data

Targeting microRNA-23a to inhibit glioma cell invasion via HOXD10

Xing Hu, Dan Chen, Yanhui Cui, Zhiyuan Li*, and Jufang Huang*

## Table of Contents:

**Supplementary Figure 1:** The whole gel blot of HOXD10 protein level in U87 cells transfected with miR-23a inhibitor and HOXD10 siRNA.

**Supplementary Figure 2:** The whole gel blot of MMP-14 protein level in U87 cells transfected with miR-23a mimic, miR-23a inhibitor and HOXD10 siRNA.

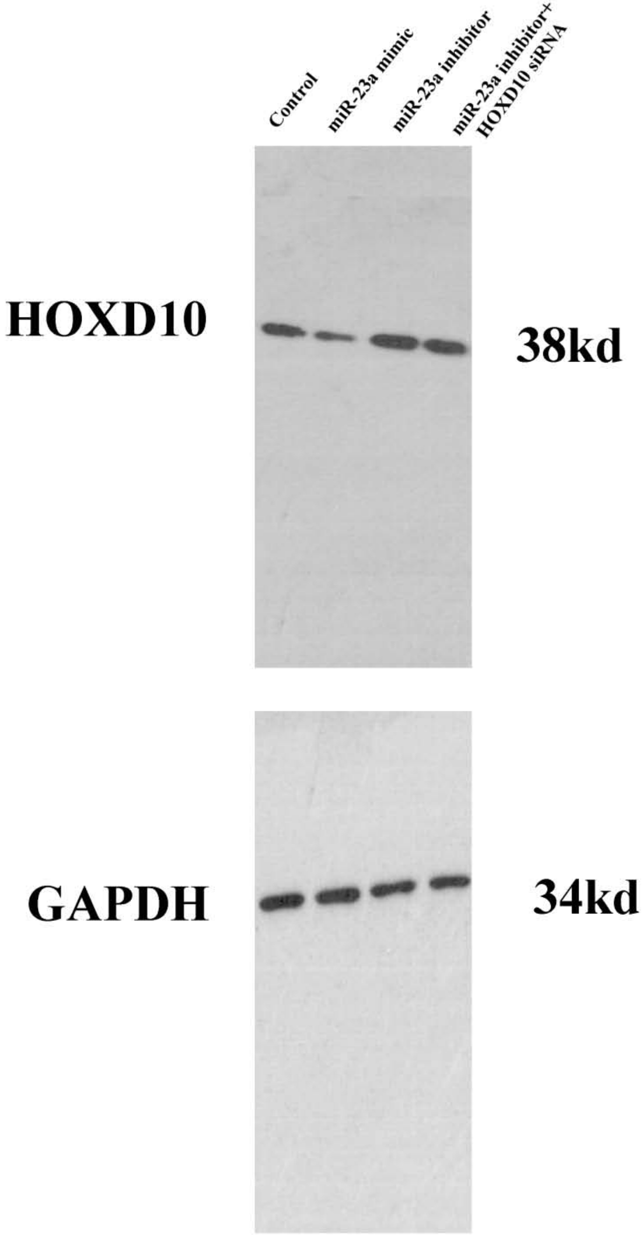


Supplementary Fig. 1. The whole gel blot of HOXD10 protein level in U87 cells transfected with miR-23a inhibitor and HOXD10 siRNA. Control: cells with mock transfection.

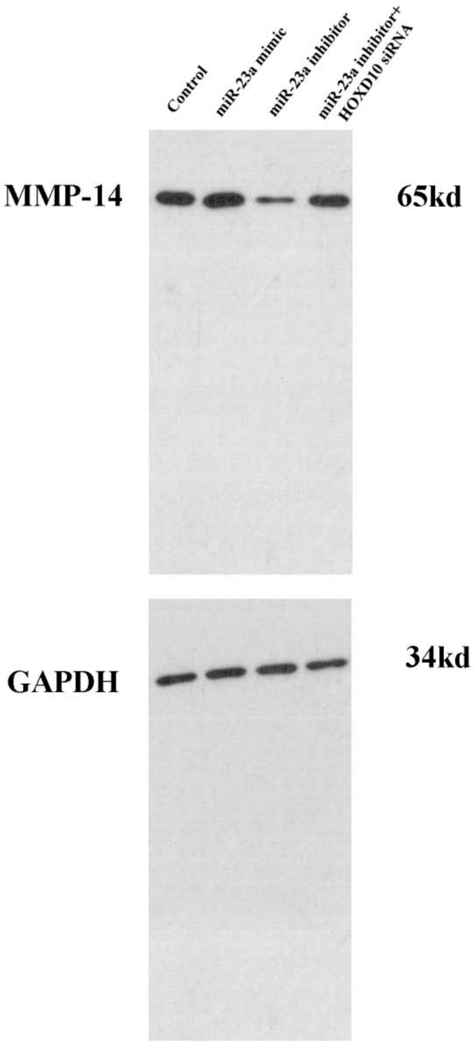


Supplementary Fig. 2. The whole gel blot of MMP-14 protein level in U87 cells transfected with miR-23a mimic, miR-23a inhibitor and HOXD10 siRNA. Control: cells with mock transfection.
